# Exploring the Role of the Gut Microbiome Across the Lifespan: Implications for Aging and Metabolic Disorders

**DOI:** 10.1210/jendso/bvaf130

**Published:** 2025-08-12

**Authors:** Anouar Aznou, Joost P H Drenth, Max Nieuwdorp, Abraham S Meijnikman

**Affiliations:** Department of Internal and Experimental Vascular Medicine, Amsterdam University Medical Centers, Location AMC, 1105 AZ Amsterdam, the Netherlands; Department of Gastroenterology and Hepatology, Amsterdam Gastroenterology Endocrinology Metabolism (AGEM), Amsterdam University Medical Center, Location VUMC, 1081 HV Amsterdam, the Netherlands; Department of Gastroenterology and Hepatology, Amsterdam Gastroenterology Endocrinology Metabolism (AGEM), Amsterdam University Medical Center, Location VUMC, 1081 HV Amsterdam, the Netherlands; Department of Internal and Vascular Medicine, Amsterdam University Medical Centers, Location AMC, 1105 AZ Amsterdam, the Netherlands; Diabetes Center Amsterdam, Amsterdam 1066 EC, the Netherlands; Department of Gastroenterology and Hepatology, Amsterdam Gastroenterology Endocrinology Metabolism (AGEM), Amsterdam University Medical Center, Location VUMC, 1081 HV Amsterdam, the Netherlands; Tytgat Institute for Liver and Intestinal Research, Amsterdam Gastroenterology Endocrinology Metabolism, Academic Medical Center, University of Amsterdam, 1105 AZ Amsterdam, the Netherlands

**Keywords:** fatty liver disease, gut microbiota, MASLD, senescence, obesity, aging

## Abstract

The gut microbiome is widely accepted to play a crucial role in human health and disease. These intestinal microbiota are not only involved in gastrointestinal physiology, but they also contribute to essential immune modulation and metabolic homeostasis. Growing evidence suggests that alterations in the gut microbiota composition are linked to various metabolic disorders, including obesity and age-related diseases. Obesity, a global public health concern, is associated with shifts in microbial diversity and functionality, which influence energy extraction, fat storage, and systemic inflammation. Similarly, age-related disorders, such as neurodegenerative diseases, sarcopenia, and metabolic syndromes are linked to gut microbiome alterations that exacerbate chronic inflammation and metabolic dysfunction. Understanding the intricate relationship between the gut microbiome, obesity, and aging-related pathologies is essential for developing targeted microbiome-based interventions to mitigate these health challenges. In this review, after a brief summary of the development of the gut microbiome across the lifespan and its modulating factors, we will focus on the mechanisms underlying the relation between the gut microbiome and metabolic and aging-related disorders. Finally, the findings of interventional studies underscoring causality and the potential future directions will be discussed, with a focus on the possibility of modifying the progression of metabolic and age-related diseases through the modulation of the gut microbiome.

The heterogeneity of human aging cannot be fully explained by genetic differences alone [1]. This observation highlights the influence of exogenous factors, such as the gut microbiota, in shaping the aging process. In recent years, the gut microbiome—the vast community of microorganisms, including bacteria, fungi, viruses, and archaea, residing in the human gastrointestinal tract—has emerged as a pivotal area of study in health and disease [2, 3]. These microbial communities dynamically interact with their host, including through the production of (diet-derived) microbial metabolites that influence metabolism, immunity, and neurological function—all of which are closely tied to aging. The gut microbiome has been taken up in the hallmarks of aging, underscoring its potential role as both a marker and driver of age-related decline [4].

Despite remarkable interindividual variability—driven by factors such as diet, lifestyle, geography, and genetics—the gut microbiome exhibits certain universal patterns in composition, diversity, and functional capacity across the human lifespan [3]. Notably, compositional and functional shifts in the microbiome have been observed in obesity and other age-related conditions, such as type 2 diabetes and its complications, including kidney disease, heart disease, and metabolic dysfunction–associated steatotic liver disease (MASLD; formerly known as nonalcoholic fatty liver disease) [5-8]. These changes underscore the gut microbiome's role in metabolic (low-grade inflammation-driven) dysregulation and systemic inflammation, both of which exacerbate aging processes.

In this review, we explore the development of the gut microbiome across the human lifespan and examine how it is shaped by factors such as diet, medication use, and environmental influences, with a particular focus on its alterations in obesity and obesity-related diseases. We further explore how these microbial changes contribute to the aging process and metabolic disorders. Finally, we look into recent intervention studies and discuss future perspectives for microbiome-targeted therapies aimed at promoting healthy aging.

## Temporal Development of the Human Gut Microbiome Across the Lifespan

The existence of a fetal microbiome remains controversial [9-12]; the first significant microbial colonization of the infant gut occurs at birth, influenced by the mode of delivery (vaginal vs cesarean). This is reflected in distinct gut microbial compositions between vaginally vs cesarean-delivered infants during their first months of life [13-15]. After delivery, the gut microbiome matures, marked by increased microbial diversity [15-19]. *Actinobacteria*, *Bacteroidetes*, *Firmicutes*, and *Proteobacteria* dominate throughout infancy and later life [15, 19-22]. Initially, facultative anaerobes and aerobes from *Proteobacteria* dominate, but strict anaerobes from the other 3 phyla become dominant as the infant matures [23-25]. Breastfeeding promotes *Bifidobacterium* colonization [21], while weaning increases *Bacteroides* and *Clostridium*, shifting the microbiome toward an adult-like composition [19, 20]. Though this transition was postulated to more or less conclude in early childhood [26], older children and even adolescents show significant differences in gut microbiome composition and lower bacterial diversity compared to adults [27-29]. Alongside these compositional changes, the maturing infant gut microbiome diversifies with alpha diversity increasing after cessation of breastfeeding and plateauing in early adulthood [30].

In adulthood, stability prevails, as described in a study of over 6500 fecal samples from healthy individuals [31]. Bacterial species richness differed significantly only between young children and other age groups, with 50 to 70 core species (prevalence ≥50%, abundance >0.1%) across most groups, except children under 4 years of age, in whom only 7 species were identified. Pregnancy may alter this, with studies reporting temporal compositional changes [32-35] and reduced alpha diversity [33-35], although others find marginal or no change [36, 37]. With advancing age, diversity declines and structure shifts. Centenarian studies show reduced *Bacteroides* and *Roseburia*, with enrichment of *Bifidobacterium* and *Akkermansia* linked to longevity [38]. These observations have been expanded by a comprehensive 2021 study analyzing gut microbiome and phenotypic data from over 9000 individuals across 3 independent cohorts, aged 18 to 101 years [39]. This analysis demonstrated that the individual gut microbial composition becomes increasingly distinct with age, a trait linked to microbial metabolites implicated in immune modulation, inflammation, and aging processes. In healthy older adults, this drift toward uniqueness persists, whereas it diminishes or stalls in those with poorer health. Notably, healthy aging is marked by a depletion of ubiquitous core taxa like *Bacteroides*, and in individuals nearing extreme age, the persistence of high *Bacteroides* abundance combined with low microbial uniqueness correlates strongly with reduced survival [39].

Contrasting findings emerge from other cohorts. The ELDERMET study [40] reported an elevated presence of core genera such as *Bacteroides*, *Alistipes*, and *Parabacteroides* in older individuals compared to younger controls, alongside age-related compositional shifts associated with frailty, cognitive decline, depression, and inflammation. Another cross-population study identified consistent age-related microbial trajectories across diverse ethnicities, noting a reduction in sex-specific microbial differences and an increased abundance of health-associated species, including *Akkermansia*, in older adults [41]. These discrepancies suggest that physiological changes tied to aging—beyond diet and lifestyle—profoundly influence gut microbiota dynamics.

The centenarian signature is marked by increased abundance of taxa such as *Clostridium*, *Methanobrevibacter*, and *Synergistetes*, while *Eubacterium rectale* and *Faecalibacterium prausnitzii* are relatively depleted [42, 43]. Interestingly, it was recently shown that centenarians possess a distinct and highly diverse gut virome, which shows close associations with specific gut bacteria such as *Alistipes* and *Clostridium* [44]. In addition, the centenarian gut harbors viruses encoding specific genes that aid bacterial production of sulfide, a compound implicated in the aging process [45].

While the gut microbiome in healthy aging drifts toward uniqueness and metabolic resilience, marked by taxa like *Akkermansia* and beneficial metabolites, this pattern is starkly absent in obesity. Across the lifespan, obese individuals exhibit a dysbiotic microbiome that deviates from the healthy aging trajectory, marked by reduced diversity, dominance of taxa such as *Bacteroides* or *Proteobacteria*, and a pro-inflammatory metabolite profile [46]. Indeed, systematic reviews highlight these differences across pediatric [47, 48], adult [47, 49] and elderly [50] populations with obesity. In children with obesity, *Eubacterium hallii* (now renamed *Anaerobutyricum soehngenii*) and *Lactobacillus* spp. were found to be decreased [48]. The former is a short-chain fatty acid (SCFA)-producer [51] that was shown to improve insulin resistance in diabetic mice [52], and a first-in-human clinical trial demonstrated a small but potentially clinically relevant improvement in glucose variability in individuals with obesity and type 2 diabetes [53]. Meanwhile, certain *Lactobacillus* species are known for their anti-obesity properties [54] that rely on their ability to decrease leptin levels and regulate lipid metabolism [55, 56]. The abundance of *Akkermansia muciniphila* is decreased in children with obesity [48], as it is in both adult and elderly obese individuals [49, 50]. Recent studies have confirmed the inverse relation between *Akkermansia* and obesity in large cohorts [57], while multiple interventional studies in mice and humans have shed a light on the potential of *A. muciniphila* as a probiotic treatment of overweight and obesity [58].

It should be noted that international cohort studies have identified differences in gut microbiome composition between the sexes, which vary across the lifespan, with sex-specific microbial divergence occurring after puberty and largely disappearing in older adults [3, 59]. These patterns suggest a role for sex hormones, supported by alterations in gut microbiome composition observed in both male and female rodents following gonadectomy (ie, removal of testes or ovaries). However, recent data from both animal and human studies indicate that the sex-specific and menopausal status-related taxonomic differences are at least partially attributable to other factors, particularly diet and age [60, 61]. In addition, another recent study investigating the role of sex differences in obesity showed that male mice gained more weight than females in response to a high-fat diet, yet this disparity remained even after antibiotic depletion of the gut microbiome [62]. This suggests that sex-dependent differences in the gut microbiome play a limited role in driving metabolic outcomes.

## Factors Influencing the “Healthy” Gut Microbiome Across the Lifespan

External factors dynamically shape the gut microbiome across life stages, influencing its composition, diversity, and functionality. Starting antenatally, examples of maternal factors that influence the composition of the infant gut microbiome include smoking or exposure to smoke [63], diet [64-66], obesity [67], and inflammatory bowel disease [68, 69]. Although cesarean-born infants show different bacterial colonization and lower abundance of important genera such as *Bacteroides* and *Bifidobacterium* [70, 71], these differences appear to be temporary and limited to the first few months of life [71]. Other early-life factors include both dietary as well as family-related environmental factors, as reviewed by Dong et al [72]. Among the most significant environmental factors influencing the infant gut microbiome is antenatal and early-life exposure to antibiotics. Bacterial diversity is lower in infants born to mothers exposed to antibiotics, and differences across multiple bacterial taxa are described with lower *Actinobacteria* and *Bacteroidetes* and higher *Proteobacteria* on the phylum level and lower *Bifidobacterium* and *Bacteroides* on the genus level [73]. Antibiotic exposure is also associated with a higher risk of obesity later in childhood [74], and longitudinal data demonstrate that multiple antibiotic courses in early childhood increase the risk of obesity later in life [75]. Interestingly, this association was not found in toddlers born from overweight mothers, while maternal body mass index (BMI) is known to influence the infant gut microbiota. Indeed, maternal pre-pregnancy BMI is associated with an altered infant microbiome at 2 days [76], 2 weeks [77], 1 month [78], 6 months [79], and 2 years of age [79].

In adulthood, the microbiome, though relatively stable, remains responsive to external inputs. Diet is a well-known modulator of the gut microbiome [80]. For example, observational and interventional data revealed that the Mediterranean diet is associated with an increase in *Faecalibacterium* [81], a genus with anti-inflammatory properties whose low levels are associated with inflammatory disorders such as inflammatory bowel disease [82]. The abundance of *Prevotella*, whose significance in human health is more ambiguous [83], also decreased with adherence to the Mediterranean diet. By contrast, the typical Western diet (high-fat, low-fiber, and ultraprocessed food) causes a gut microbiome marked by low microbial diversity, and a decrease in taxa such as *Bacteroidetes* and *Bifidobacteria* [84]. Mice fed a Western-style diet demonstrated an increase in fecal inflammatory markers and decrease in SCFA levels, with a microbiome marked by lower bacterial diversity and species richness and high abundance of pro-inflammatory species belonging to *Proteobacteria* [85]. Interventions involving dietary fiber lead to increases in the abundance of the genera *Bifidobacterium* and *Lactobacillus*, although they do not change gut microbial diversity [86]. In addition, numerous interventional studies show that a low carbohydrate diet is associated with taxonomical alterations, including lower relative abundance of *Firmicutes* and *Bifidobacterium*, yet higher *Bacteroidetes* abundance.

In addition to diet, physical exercise has been identified as another possible modifier of the adult microbiome. Two recent meta-analyses yield conflicting results on the association between exercise interventions and changes in bacterial (alpha) diversity and taxonomical compositions of the gut microbiome in adults [87, 88]. One systematic review demonstrated a positive association between exercise interventions and alpha diversity [87], whereas the other only supported this positive association for prolonged exercise and cardiorespiratory fitness while failing to demonstrate this for short-term exercise interventions [88]. In addition, while the first analysis found a significant reduction of *Bacteroidetes* abundance and a significant increase in *Firmicutes* abundance [87], the other refrained from drawing firm conclusions in view of heterogeneity from the results of the included studies [88]. However, both studies agreed on the mediating role of short-chain fatty acids (SCFAs), bacterial metabolites linked to improved glucose metabolism [89], pointing to the positive association between exercise and both SCFAs as well as specific SCFA-producing microbiota.

Yet another modulating factor of the adult gut microbiome is altitude [90]. Recent data from longitudinal studies involving healthy Chinese men who migrated from and toward a higher latitude demonstrate the profound effect of a change in altitude on the composition and function of the gut microbiome [90, 91]. In these individuals, high altitude was associated with a decrease of α-diversity and an increased abundance of anaerobes such as *Blautia* and *Prevotella* was observed, as well as alterations of multiple functional metabolic pathways.

In the elderly, one of the most critical factors influencing the gut microbiome is their living arrangement, that is, whether they are institutionalized or community-dwelling. The gut microbiome of elderly individuals can be clustered based on their housing arrangement, with the microbiota of long-time institutionalized elderly being significantly more divergent from younger controls as compared to the microbiome of their community-dwelling peers [92]. In addition, the gut microbiome of long-term institutionalized elderly is marked by significantly lower diversity and metagenomically fewer microbial genes related to SCFA-production. It remains unclear whether these institutionalization-associated signatures are reflective of a frailty phenotype or are mainly driven by environmental changes occurring upon institutionalization, such as dietary changes, as some studies suggest [93].

Irrespective of age, both antibiotic and non-antibiotic drugs can profoundly alter the composition and functionality of the gut microbiome. Short-course antibiotic treatments cause significant yet generally transient disruptions in microbial diversity and composition, depending on multiple factors of the host and microbiome itself [94, 95]. This is exemplified by patients undergoing *Helicobacter pylori* eradication therapy, in whom recovery of the gut microbiome is generally observed within 1 year following this aggressive antibiotic course [96]. Similarly, certain non-antibiotic drugs, such as proton pump inhibitors and antidiabetics, can mimic the effects of antibiotics on the microbiome [97]. These effects are of particular importance in the context of prolonged use and polypharmacy, which are common among elderly populations [98]. A large Italian cohort of acutely hospitalized elderly individuals possessed a gut microbiome with reduced species richness and distinct dysbiotic bacterial composition compared to nonhospitalized elderly controls without polypharmacy. In the hospitalized cohort, a significant association between polypharmacy of dysbiosis and polypharmacy was found, which was independent of other multimorbidity parameters [99]. Similar results were found in long-term residents of nursing homes, in whom non-antibiotic medications were strong independent predictors of gut microbiome composition [100].

Given the nature of polypharmacy, little is known about the reversibility of these polypharmacy-associated changes in the microbiome. However, the dramatic changes in gut microbiota observed in polypharmacy-exposed mice were largely reversed upon cessation of medication [101].

While all these factors shape the microbiome, certain influences—particularly polypharmacy, antibiotics, and Western diets—disrupt diversity and functionality in ways resembling dysbiosis, promoting pro-inflammatory states often absent in healthy aging. In contrast, factors like exercise and Mediterranean diets support microbial resilience, mirroring patterns seen in healthy longevity. This divergence likely stems from the production of microbial metabolites with signaling properties, which will be explored in the next section.

## From Composition to Functionality

**Figure 1.**
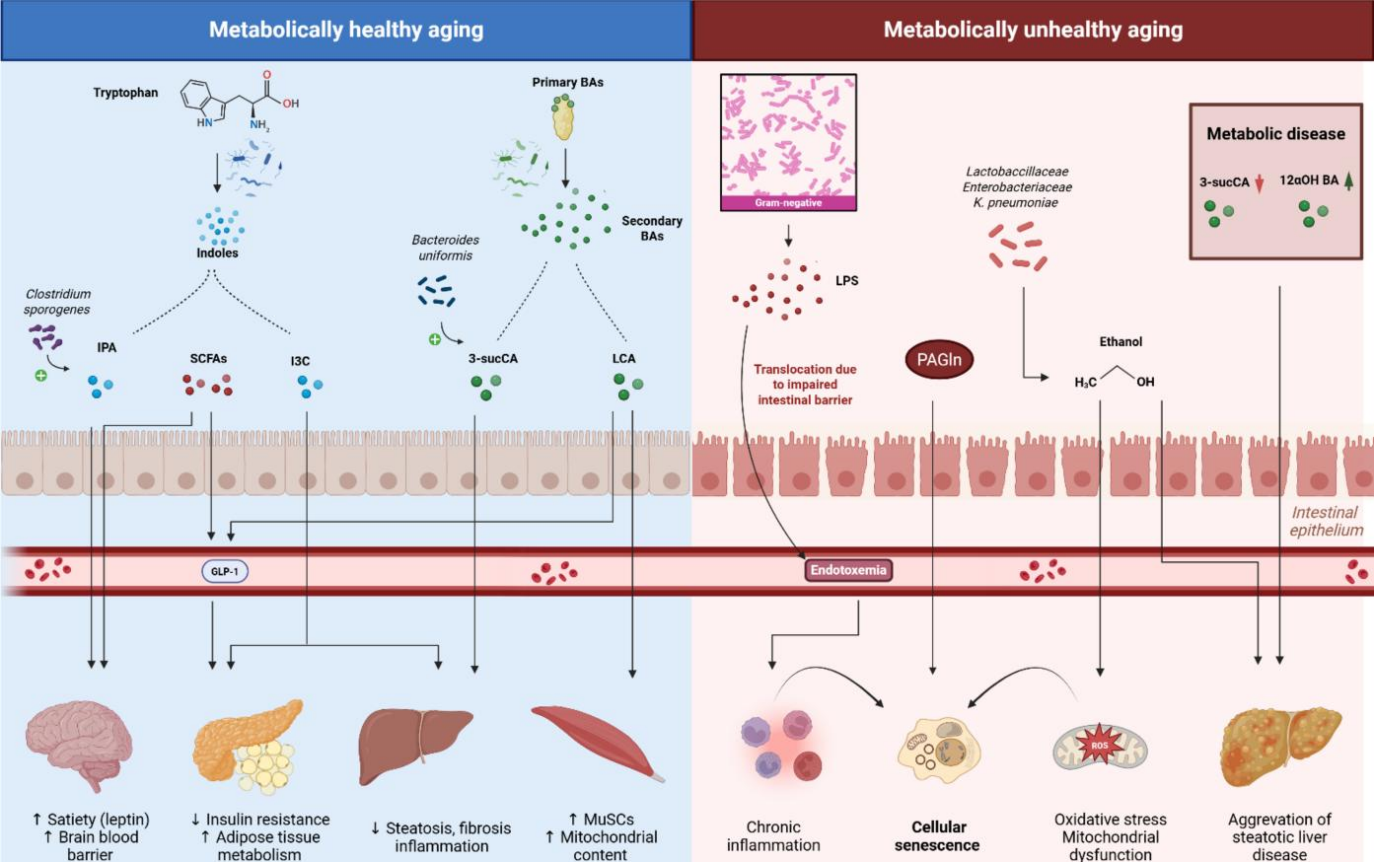
Overview of various microbial metabolites and their effects on metabolic and aging-related processes.

Abbreviations: 12aOH, 12 alpha-hydroxylated; 3-sucCA, 3-succinylated cholic acids; BAs, bile acids; GLP-1, glucagon-like peptide-1; I3C, indole-3-carbinol; IPA, indole-3-propionic acid; LCA, lithocholic acid; LPS, lipopolysaccharides; MuSCs, muscle stem cells; PAGln, phenylacetylglutamine; ROS, reactive oxygen species; SCFAs, short-chain fatty acids.

The gut microbiome exerts a profound influence on host physiology, aging, and metabolic health—primarily through its functional output rather than taxonomic composition. Microbial metabolites act as key signaling molecules, regulating inflammation, energy balance, and cellular repair. Across aging studies, metabolite profiles tend to be more consistent than microbial taxa, underscoring the importance of functionality in shaping health span and disease susceptibility (Fig. 1).

Age-related microbial shifts align with several “Hallmarks of Aging” proposed by López-Otín et al [4], particularly “altered intercellular communication” and “disabled macroautophagy.” Microbial diversity supports the generation of health-promoting metabolites like SCFAs, indoles, and bile acids, which modulate immune function and maintain cellular homeostasis [102]. In healthy aging, increased microbial uniqueness and a reduction in core taxa such as *Bacteroides* are associated with anti-inflammatory compounds that promote autophagy and metabolic resilience [103]. In contrast, reduced diversity and sustained *Bacteroides* dominance correlate with higher levels of harmful metabolites such as p-cresol, which impair signaling and contribute to aging-related decline [103].

Alterations in the microbiome seen in obesity shift metabolic outputs, favoring the production of compounds such as endogenous ethanol. Ethanol disrupts mitochondrial function, promotes oxidative stress, and has been implicated in the acceleration of aging phenotypes [104]. Similarly, increased levels of lipopolysaccharide (LPS)—a component of gram-negative bacterial membranes—can enter systemic circulation due to compromised intestinal barrier function. This low-grade endotoxemia contributes to chronic inflammation and is linked to age-related and metabolic diseases such as diabetes and MASLD [105].

Disruption of gut barrier integrity further connects the microbiome to liver pathology via the gut-liver axis. When intestinal permeability increases, microbial metabolites and endotoxins leak into the portal circulation, reaching the liver and activating inflammatory pathways that contribute to steatosis and fibrosis [106]. Meta-analyses confirm significantly higher intestinal permeability in both pediatric and adult MASLD patients, which worsens with disease progression [107].

While taxonomic diversity provides a structural overview of the microbiome, its functional capacity, especially metabolite production, more accurately reflects its impact on aging [108]. Within the framework of altered intercellular communication, these microbial metabolites serve as messengers linking gut activity to systemic physiological processes [109, 110]. Understanding their biosynthesis and biological effects is essential to unraveling the microbiome's role in longevity and age-related disease.

### Bile Acids

Bile acids are among the most significant microbiota-derived metabolites. Long known for their role in lipid digestion and therapeutic potential in gallstones, it was not until the early 2000s that these cholesterol-derived steroids were identified as ligands for various nuclear receptors such as the farnesoid X (FXR), vitamin D receptor (VDR), pregnate x receptor (PXR), and constitutive androstane receptor (CAR) [111]. Through the signaling pathways downstream of these receptors, bile acids influence a wide range of metabolic processes, including lipid, glucose, and drug metabolism. Accordingly, bile acids are implicated in metabolic disorders such as obesity and MASLD [112].

Bile acids show a bidirectional relationship with the intestinal microbiota. Despite their bacteriotoxic properties, bile acids are known to support microbial diversity by altering gut microbiome composition [113]. Conversely, gut bacteria regulate hepatic bile acid synthesis and synthesize enzymes crucial for the biotransformation of primary conjugated bile acids to secondary bile acids [114].

In mice, administration of lithocholic acid (LCA) ameliorated aging-related phenotype with improvement of both muscle and metabolic function. LCA was shown to induce muscle stem cells and increase mitochondrial content and respiratory function of muscle tissue, while improving insulin resistance and increasing serum glucagon-like peptide 1 (GLP-1) levels [115]. These effects are explained by the ability of LCA to activate the protein kinase AMPK, a “master regulator” of energy homeostasis that also plays an important role in regulating various aging-associated signaling pathways [116].

In humans, there is a positive correlation between BMI and total plasma bile acids [117]. In people with obesity there is a shift in the synthetic pathways of bile acids toward 12α-hydroxylated bile acids compared to lean individuals [118], and this change is also seen in metabolically unhealthy obese vs healthy obese counterparts [119]. However, data on differences in bile acid levels and compositions in humans remain scarce and should be interpreted with caution given high interindividual variety and discrepancies between portal vein and peripheral bile acid pools [112]. Indeed, it was recently shown that bile acid levels in patients with severe obesity were 7-fold higher in the portal vein compared to peripheral levels [120].

A recent study characterized the secondary bile acid 3-succinylated cholic acid and its synthesization by an enzyme expressed in *Bacteroides uniformis* [121]. This bile acid, which is depleted in MASLD patients, was shown to decrease hepatic steatosis, fibrosis, and inflammation by directly promoting the growth of *A. muciniphila*, while suppressing *Clostridium sporogenes* and *Enterococcus hirae.*

New research in the past years has uncovered a suite of biosynthetic enzymes—such as those driving 5α-reduction, 7α-dehydroxylation, and 24-reconjugation—that mediate microbial bile acid transformation [42, 122]. These processes may generate a plethora of unique bile acid structures, underscoring the complexity of microbial metabolism and signaling a need to redefine the intricate interplay between the gut microbiota and its host in shaping health and aging.

### Short-Chain Fatty Acids

Numerous bacterial species inhabiting the gut are involved in the fermentation of fiber, which leads to the production of SCFAs, the most important of which are acetate, butyrate, and propionate. SCFA levels vary across the lifespan, increasing from infancy to adulthood and then declining again in elderly adults [123, 124]. These dynamics are reflective of the changes in the abundance of specific SCFA-producing taxa that occur over the lifespan [125-127]. SCFAs are ligands of receptors, most important of which belong to the G-protein-coupled receptors (GPR) family, expressed by various cells throughout the body. This explains the broad range of processes in which SCFAs are involved, including their endocrine, immunological, and neuronal effects. At the local level, SCFAs contribute to mucosal and epithelial integrity and immunity by serving as energy source for enterocytes, promoting epithelial barrier function and dampening pro-inflammatory pathways in immune cells [128]. Importantly, SCFAs are involved in systemic energy homeostasis by stimulating enteroendocrine cells to produce hormones involved in satiety, such as GLP-1 and leptin, while simultaneously influencing adipose tissue metabolism [129]. Furthermore, there is mounting evidence for the involvement of SCFAs in the “gut-brain axis,” including through their ability to reinforce the blood-brain barrier by stimulating the expression of tight junction proteins [130].

Early observations of elevated fecal SCFA levels in obese individuals led to the hypothesis that the shift in these levels are caused by obesity-related taxonomical changes in microbiota, for example the presumed altered F/B ratio [131]. However, the suggested mediating role of SCFA levels in the link between obesity and gut microbiome is not without controversy. A recent meta-analysis confirmed elevated fecal and serum concentrations of the 3 main SCFAs in obese vs non-obese individuals but failed to demonstrate phylum-level differences in microbiome composition [132]. Similarly, another recent study found that while SCFA levels were predictive for obesity in a large multinational cohort, this relationship was not mediated by gut microbiota diversity [46].

### Amino Acids

Gut microbiota synthetize and metabolize several essential amino acids, notably tryptophan, which is converted to major metabolites such as serotonin, kynurenine, and indoles. Obesity alters tryptophan metabolism, shifting it away from the indole pathway as indicated by a depletion in serum indole levels in both obese children and adults compared to lean controls [133, 134].

Conversely, indoles exhibit anti-obesity effects. For example, it was recently shown that indole-3-carbinol (I3C) induced weight reduction in obese mice, reducing insulin resistance, hepatic steatosis, and systematic inflammation markers while increasing beneficial gut bacteria like *Akkermansia* and *Eubacterium* and restoring the intestinal barrier [135]. Similarly, indole-3-propionic acid (IPA) showed anti-obesity effects in diet-induced but not genetic obesity, explained by an IPA-mediated increase of sensitivity to leptin, with *C. sporogenes* boosting IPA levels and reversing weight gain [136].

Germ-free mice colonized with an *E. coli* strain incapable of converting tryptophan to indoles exhibited impaired glucose metabolism and increased food intake, lipolysis, and oxidative stress, yet paradoxically lost weight due to gastrointestinal dysfunction mimicking age-related decline [137].

Indeed, indoles are implicated in aging through their action on the aryl hydrocarbon receptor (AHR), a transcription factor involved in xenobiotic and energy metabolism, inflammation, and aging [138, 139]. Through Ahr-dependent pathways, microbiota-derived indoles increased the health span of both young and aged mice, and alleviated age-related pathological phenotypes in rodents, such as stroke [140] and osteoarthritis [141]. However, the role of the aryl hydrocarbon receptor (AHR) in the aging process remains ambiguous, with evidence suggesting both pro- and anti-aging effects depending on context and experimental conditions [139].

Another gut microbiome-derived metabolite linked to aging is phenylacetylglutamine (PAGln). Yang et al [142] showed that circulating PAGln levels increase with age and correlate with specific microbial signatures in elderly individuals. In addition, they demonstrated that PAGln induced cellular senescence, a hallmark of aging defined as a cell state marked by cell cycle arrest [4], both in vitro and in vivo.

## Interventions

### Diet

The connection between the gut microbiome and healthy aging suggests that it may be a modifiable factor in preventing or slowing health decline in older adults, similar to the connection between dysbiosis and obesity. However, interventions targeting the microbiome have yielded mixed results. Among dietary strategies, the most notable is the “Nu Age” trial—a year-long, multicenter study in community-dwelling older adults that tested the impact of a Mediterranean diet [143]. The intervention led to beneficial shifts in the microbiome, including an increase in species such as *F. prausnitzii*, which were associated with improvements in objective frailty measures like grip strength and neuropsychiatric scores. While encouraging, these effects were largely limited to disease-oriented metrics, with no significant improvement in cognition [144]. These findings highlight both the potential and the limitations of dietary interventions in modulating the microbiome for healthy aging.

### Prebiotics

A prebiotic is defined as a substrate which exerts health benefits on the host after its utilization by host microbiota [145]. Common prebiotics include inulin, lactulose, and various types of oligosaccharides [146]. Inulin, a soluble dietary fiber, is known to promote beneficial microbiota like *Bifidobacterium*, *F. prausnitzii*, and *Lactobacillus [*147]. Its fermentation by gut microbiota releases SCFAs, which exert a range of metabolic effects as discussed previously. While it does not appear to significantly impact body weight [147], a recent placebo-controlled trial in children with obesity showed increased muscle-building biomarkers after 6 months of inulin [148]. In vitro, inulin downregulated pro-inflammatory genes (IL-1β, IL-6, TNF) in macrophages, while upregulating anti-inflammatory markers (TGF-β, FIZZ-1). However, a 12-week inulin intervention in elderly twins showed no improvement in muscle strength or function [149].

Recently, resistant starch, which evades small intestine digestion and is fermented in the colon, has emerged as a promising prebiotic. In a large randomized controlled trial, a 4-month resistant starch intervention in MASLD patients significantly improved hepatic steatosis and lipid profile and reduced body weight, BMI, adipose tissue, and systemic inflammatory markers [150]. Another randomized controlled trial in overweight and obese individuals found similar beneficial results on body composition and improvement of insulin resistance in the resistant starch-treated group [151]. Resistant starch–induced changes in microbiota and metabolites played a key role in both studies, with its anti-obesity effects being mediated through increased *Bifidobacterium adolescentis* and a reduction in *Bacteroides stercoris* and serum levels of branched-chain amino acids, both directly linked to MASLD progression [150, 151].

### Probiotics

Probiotics are defined as live microorganisms that provide health benefits to the host when administered adequately [152]. The predominant probiotic strains present in food and dietary supplements are *Bifidobacterium* and *Lactobacillus*, although various bacterial strains have been ascribed probiotic properties. While probiotics clinically are recommended for certain gastrointestinal disorders [153], they are being studied for their potential therapeutic role in a wide range of conditions, including obesity and age-related conditions.

In a 2025 systematic review and meta-analysis which included 8 randomized controlled trials using oral probiotics in obese individuals, the probiotic-treated group experienced significantly more weight loss, decrease in waist circumference, and reduction of visceral fat [154]. Paradoxically, no such effect was found for BMI.

In mice, oral administration of *A. mucinphila* in mice caused an increase of intestinal levels of anti-aging metabolites including SCFAs and various bile acids [155], counteracted the age-related decline of the colonic mucus barrier and dampened pro-inflammatory signaling in the colon [156]. In accelerated-aging mouse models, strains of *Lactobacillus* reversed induced oxidative stress [157, 158] and attenuated loss of muscle mass and strength [158]. Similar anti-sarcopenic effects have been found in humans. Two recent systematic reviews have demonstrated significant positive effects of probiotic supplementation on muscle strength and muscle mass in elderly populations [159, 160], pointing toward a possible positive role of probiotics in the setting of frailty and sarcopenia. Numerous clinical trials are underway exploring the broader anti-aging potential of these next-generation probiotics [161].

### Postbiotics

Postbiotics contain lifeless microorganisms or microbial components—such as inactivated bacterial cells, cell wall fragments, metabolites, or other probiotic-derived compounds—that exhibit health benefits on the host [162]. Unlike probiotics, which require live bacteria to exert effects, postbiotics offer a nonviable alternative that can still positively influence host physiology, making them an appealing option for therapeutic applications.

Preclinical data shows that postbiotic treatments can reduce body and adipose tissue weight through various mechanisms, including regulating lipid metabolism genes, reducing oxidative stress, and downregulating pro-inflammatory gene expression [163]. For instance, postbiotics are shown to upregulate genes like PPAR-α (peroxisome proliferator-activated receptor-alpha), which promotes fat breakdown, suppresses lipogenic pathways that contribute to fat accumulation, and help mitigate cellular damage caused by reactive oxygen species, a factor often linked to obesity and metabolic disorders. They also lower levels of pro-inflammatory cytokines like TNF-α and IL-6, helping to counter inflammation-driven weight gain. Moreover, heat-killed *B. adolescentis* improved colonic senescence in mice by inducing proliferation of intestinal stem cells, downregulating senescence markers such as p21 and p53, and blocking the Wnt/β-catenin pathway in colonocytes [164]. This suggests that postbiotics may counteract age-related decline in gut function by promoting tissue regeneration and maintaining intestinal barrier integrity. The suppression of p21 and p53, key regulators of cellular aging, indicates a potential anti-senescence effect, while inhibition of the Wnt/β-catenin signaling pathway—a mechanism often dysregulated in cancer and aging—highlights a broader protective role in gut health.

### Fecal Microbiota Transplantation

Fecal microbiota transplantation (FMT) refers to the transfer of stool from a healthy donor to a recipient through oral capsules, enemas, gastrointestinal tubes, or endoscopy. While currently used mainly to treat recurrent *Clostridioides difficile* infections, emerging murine studies suggest FMT may have therapeutic potential for metabolic and age-related disorders.

Studies have demonstrated that the aging phenotype and associated diseases can be transferred to young recipients through FMT from aged animals, whereas transplanting microbiota from young to aged mice improves aging-associated traits. For example, FMT from aged to young mice induced translocation of microbiota-derived products and increased systemic inflammation (inflammaging) [165, 166]. Conversely, FMT from young to aging animals showed numerous beneficial effects, including improved glucose sensitivity and epithelial barrier function, and reduced systemic inflammation, hepatic injury, and gut dysbiosis [167]. Supplementation with acetate and the SCFA-producer *A. muciniphila*—both reduced in aged mice—yielded benefits similar to those of FMT [167].

As summarized by Novelle et al, numerous clinical trials have been published or are underway exploring the potential role of FMT in metabolic and aging-related disorders, including type 2 diabetes, metabolic syndrome, MASLD, frailty, and neurodegenerative diseases. So far, results of randomized controlled trials that examine the effect of FMT on weight loss are mixed: several found no effect on weight in obese individuals [168-170], while others reported a reduction in abdominal adiposity compared to placebo [171]. Another study observed less regain of weight and waist circumference in obese individuals after autologous FMT combined with a high-polyphenol Mediterranean diet [172].

In terms of insulin resistance, a hallmark of obesity, Vrieze et al [173] reported improved insulin sensitivity after allogenous FMT in obese patients, and several recent trials have validated this finding [174-176]. Post-FMT outcomes on lipid profiles have been inconsistent across studies [169-171, 177].

Similarly, conflicting results were found between randomized controlled trials investigating the effect of FMT in patients with MASLD. While one study observed reduced elastographically measured hepatic steatosis in MASLD patients after FMT compared to probiotic treatment [178], another trial showed no histopathological effect despite significantly altered hepatic gene expression [179]. In accordance with the latter, a third randomized controlled trial demonstrated no significant changes in magnetic resonance imaging–based liver fat quantification after FMT [180].

## Future Perspectives

Growing preclinical and translational evidence supports a causal role for the gut microbiome in aging, obesity, and related metabolic disorders among others through the production of bioactive microbial metabolites. These compounds exert wide-ranging effects on host physiology, influencing immune signaling, metabolic regulation, mitochondrial function, and cellular maintenance. In doing so, they intersect with biological processes that are central to aging and contribute to the development of conditions such as insulin resistance, MASLD, and systemic low-grade inflammation.

However, translating this mechanistic insights into clinically meaningful interventions remains challenging. A central question is how to define success: are improvements in surrogate biomarkers—such as epigenetic clocks or inflammatory profiles—sufficient, or must benefits be confirmed through long-term randomized trials showing delayed disease onset, improved physical or cognitive function, or increased health span? Similar questions apply to obesity and metabolic disease: should microbiome changes be considered therapeutic only if they lead to sustained improvements in glycemic control, liver health, or body composition?

Future research should prioritize the development of more targeted and individualized microbiome interventions tailored to a person's microbial profile, diet, genetics, and metabolic state. Integrating multi-omics technologies—metagenomics, metabolomics, and transcriptomics—will be key to identifying predictive microbial signatures and therapeutic targets.

Innovative strategies, such as engineered probiotics (Table 1), postbiotics, and targeted delivery of microbial metabolites, may offer more controlled and reproducible ways to modulate host-microbiome interactions than probiotic or dietary interventions. Ultimately, progress hinges on further unraveling specific microbial pathways that influence host aging and metabolism. As we gain deeper insights into how gut-derived signals regulate immune aging, nutrient sensing, mitochondrial health, and tissue repair, we will be better positioned to design interventions that move beyond correlation and toward causation. With robust biomarkers, mechanistic clarity, and well-designed clinical trials, microbiome-based therapies may become a powerful tool in the prevention and treatment of aging-related and metabolic diseases.

**Table 1.** Examples of recent studies investigating the effect of engineered probiotics in metabolic and aging-related diseases

Type of engineered probiotic	Application in disease (model)	Subject species	Results	Article
Secretion of IL-22 by *Lactobacillus reuteri*	Obesity and MASLD	Mice (HFD)	↓ liver weight and hepatic TG levels	Oh et al (2020) [181]
Expression of human proinsulin and human IL-10 by *Lactococcus lactis*	Type 1 diabetes	Humans	Adults: significant ↓ of C-peptide at 12 months; significant ↓of HbA1c at 3 and 6 months	Mathieu et al (2023) [182]
			Adolescents: significant ↓ of C-peptide at 6 months	
Production of GLP-1 by *Lactobacillus plantarum*	Type 2 diabetes	Mice (HFD/STZ and db/db)	↓ weight, fasting blood glucose, expression of pro-inflammatory markers in pancreatic tissue ↑ glucose tolerance	Hu et al (2023) [183]
Butyric acid production by *Bacillus subtilis* SCK6	Obesity	Mice (HFD)	↓ body weight, body weight gain, food intake, serum TC, TG, and ALT	Bai et al (2020) [184]
Consortium of butyrate-producing *Bacillus subtilis* SCK6 and *Bifidobacterium pseudocatenulatum JJ3*	Obesity	Mice (Ob/Ob)	↓ body weight gain, fasting glucose, HOMA-IR, liver weight, serum TBA, ALT, AST, ALP, TC, LDL, and HDL	Chen et al (2023) [185]
GLP-1 production by *Lactococcus lactis* MG1363	Obesity	Mice (HFD)	↓ body weight gain, adipose tissue weight, serum and hepatic TG, hepatic steatosis	Wang et al (2021) [186]
Secretion of GLP-1 analog by *E. coli Nissle 191*	Obesity	Mice (HFD)	↓ body weight, body weight gain, food intake, adipose tissue weight, liver weight ↑ glucose tolerance	Ma et al (2020) [187]
Expression of GLP-1 by *Lactococcus lactis*	AD and PD	Mice	↓ memory impairment and motor dysfunction	Fang et al (2020) [188]
Expression of human p62 protein by GM *Lactococcus lactis*	AD	Mice	↑ memory performance ↓ reduction of inflammation and amyloid peptides load in the brain	Cecarini et al(2020) [189]

Abbreviations: AD, Alzheimer's disease; ALT, alanine transaminase; AST, aspartate transaminase; HDL, high-density lipoprotein; HOMA-IR, homeostatic model assessment of insulin resistance; HFD, high-fat diet; LDL, low-density lipoprotein; PD, Parkinson's disease; TBA, total bile acid; TC, total cholesterol; TG, triglycerides.

↑ = higher or increase/improvement.

↓ = lower or decrease.

## Data Availability

Not applicable.

## Abbreviations

AHR: aryl hydrocarbon receptor
BMI: body mass index
FMT: fecal microbiota transplantation
GLP-1: glucagon-like peptide 1
LCA: lithocholic acid
MASLD: metabolic dysfunction–associated steatotic liver disease
SCFA: short-chain fatty acid
